# Technostress, psychosocial safety climate, and worker well-being in automated manufacturing: A two-wave study

**DOI:** 10.1371/journal.pone.0345249

**Published:** 2026-04-03

**Authors:** Umut Elbir, Kagan Cenk Mizrak

**Affiliations:** 1 Department of Occupational Health and Safety, Istanbul Bilgi University, Istanbul, Turkiye; 2 Department of Business Administration, Nisantası University, Istanbul, Turkiye; Dong-A University College of Business Administration, KOREA, REPUBLIC OF

## Abstract

The rapid digitalization of manufacturing has introduced new psychosocial risks alongside productivity gains, particularly in the form of technostress. This study investigates how technostress influences worker mental health, burnout, engagement, and safety outcomes in automated manufacturing environments, and whether job resources and psychosocial safety climate (PSC) serve as protective factors. Drawing on the Job Demands–Resources (JD-R) model, a two-wave panel design was employed with data collected from 605 employees at baseline and 450 employees at a four-week follow-up across five automated manufacturing plants. Multilevel and moderation analyses revealed that while direct effects of technostress on outcomes were directionally consistent with theoretical expectations, they were not statistically significant. Instead, job resources—including supervisory ICT support, recovery opportunities, and digital literacy—emerged as significant buffers, mitigating the potential negative effects of technostress. Furthermore, PSC at the crew and organizational level predicted improved engagement, better mental health, and stronger safety behaviors, underscoring its role as a higher-order organizational resource. These findings suggest that technostress in manufacturing is contingent on contextual factors: it becomes harmful when resources are insufficient and protective climates are absent. The study contributes to technostress and occupational health literature by integrating psychosocial and technological stressors in a multilevel framework and offers practical guidance for managers seeking to balance digitalization with worker well-being.

## Introduction

The accelerating digitalization of manufacturing has reshaped both the technical and psychosocial fabric of work, as collaborative robots, digital quality monitoring, and ICT-enabled workflows diffuse across lines and shifts and alter daily routines, performance expectations, and the cadence of learning. These technologies create opportunities for efficiency and innovation, yet they also introduce continuous digital demands, algorithmic oversight, and rapid cycles of adaptation that can overwhelm workers’ coping resources and exhaust attentional bandwidth in safety-critical environments. In this context, technostress—a cluster of ICT-related stressors such as techno-overload, techno-invasion, and techno-complexity—has emerged as a salient occupational risk with consequences for mental health, burnout, engagement, and safety performance [1,2]. While technostress has been widely studied in service or ICT-intensive settings, recent research shows it is equally consequential in frontline operational roles, including hospitality, food production, and banking, indicating that production workers interacting with robotics and AI-driven systems are similarly vulnerable [3]. These developments heighten the need to understand how technostress unfolds within automated manufacturing, where process deviations have safety implications, throughput logics intensify digital demands, and human–machine teaming makes individual strain a systems-level concern that reverberates through quality, reliability, and organizational resilience.

Despite this importance, evidence in heavy manufacturing remains limited and methodologically narrow, with many studies relying on single-wave, cross-sectional designs that capture only a snapshot of workers’ experiences and thereby obscure temporal ordering, adaptation, and the differentiation between transient strain and persistent risk. Prior work also seldom separates person-level processes from group- or line-level climates, even though production occurs in nested social units where shared priorities and safety norms condition both perceived techno-demands and the translation of strain into health, motivation, and safety behaviors. At the same time, research has not systematically assessed how job resources—such as supervisory ICT support, recovery opportunities, and digital literacy—interact with techno-demands to shape downstream outcomes, nor how an upstream organizational climate like Psychosocial Safety Climate (PSC) may alter the exposure–response relationship by signaling management’s commitment to psychological health, participation, and learning [4,5]. Anchored in the Job Demands–Resources (JD-R) framework, the purpose of this study is therefore to investigate whether and how technostress relates to worker mental health, burnout, engagement, and safety in automated manufacturing, and to test the protective roles of ICT-specific and general job resources, as well as PSC, within a time-lagged, nested design that reflects how manufacturing is actually organized on the shop floor.

H1: Higher technostress will lead to worse mental health, greater burnout, lower engagement, and poorer safety (including more near-misses) over time.

H2: Stronger job resources—both ICT-specific (e.g., involvement facilitation, ICT control/support) and general (e.g., supervisory support, recovery opportunities, digital literacy)—will weaken (buffer) the negative effects of technostress on these outcomes.

H3: A stronger Psychosocial Safety Climate will be associated with lower technostress and better worker outcomes, while also enhancing the protective (buffering) impact of job resources against techno-demands.

Relative to prior work, the novelty of this paper lies in four complementary advances that extend scope conditions for technostress theory and make the findings more actionable for digitally intensive manufacturing. First, we implement a two-wave panel in automated manufacturing rather than relying on cross-sectional snapshots from service or IT settings, strengthening temporal precedence and external validity for frontline production contexts where human–machine interactions and shift structures are distinctive and where recovery dynamics unfold over time. Second, we jointly model technostress creators with a multi-criterion outcome set—mental health, burnout, engagement, and safety/near-miss-related behaviors—to reflect the multidimensional performance and risk profile of high-reliability operations, an integration rarely attempted in this domain yet essential for capturing trade-offs managers face. Third, we differentiate ICT-specific from general resources and test their comparative buffering value, offering concrete levers that range from tool-level supports to supervisory practices and recovery design, thereby translating JD-R logic into operational interventions that are feasible under throughput and cost constraints. Fourth, we treat PSC as an upstream, potentially multilevel resource, addressing the underexplored question of how shared psychosocial priorities at the crew/line level shape individual strain and safety-relevant behavior in automated plants and clarifying how climate can amplify or dampen the payoffs from local resource investments [4,5]. Finally, we situate these micro-level contributions within a macroeconomic and information-dynamics context, aligning with evidence that well-being–relevant infrastructure co-moves with regional growth [6] that fractional/information-theoretic approaches reveal persistent, directional information flows shaping economic interactions [7,8] that distributionally robust decision frameworks under ambiguity have policy relevance for performance and resilience [6]. By explicitly stating purpose, objectives, and hypotheses, and by articulating these four contributions with a macro-aligned perspective, the introduction clarifies what is tested, why it matters, and how the study adds novelty beyond existing research on technostress in manufacturing and related sectors.

To align the narrative with these aims, the remainder of the paper reviews technostress, job resources, and PSC within the JD-R framework and details the measures and setting, then reports multilevel, time-lagged estimates for main effects and moderation by resources alongside PSC’s role as an upstream context, and finally discusses implications for theory and practice in digital manufacturing. This structure ensures that the confirmatory intent is transparent from the outset, that the analytic strategy fits the nested realities of shop-floor work, and that the resulting evidence can inform targeted interventions—balancing throughput with worker well-being—at both the tool/process level (ICT supports, recovery design) and the climate level (strengthening PSC) in organizations navigating the pressures and promises of Industry 4.0

## Literature review & theoretical framework

### Technostress in industry

The concept of technostress was formally conceptualized by Tarafdar, Tu, Ragu-Nathan, and Ragu-Nathan (2007), who defined it as the stress that employees experience due to the challenges of using information and communication technologies (ICTs). Their influential framework identified five key “technostress creators”: techno-overload, where technology forces individuals to work faster and longer; techno-invasion, which blurs the boundaries between work and private life; techno-complexity, reflecting the difficulties of learning new systems; techno-insecurity, the fear of job loss due to others’ superior technological skills; and techno-uncertainty, the stress associated with rapid and constant technological changes. These dimensions were shown to increase role stress and reduce productivity, thereby making technostress not only an individual well-being issue but also an organizational performance concern. Later work by Stadin et al. [2], using the Swedish Longitudinal Occupational Survey of Health (SLOSH), operationalized technostress as ICT demands across occupational groups. Their findings demonstrated that ICT-related demands consistently predicted psychosocial strain among managers and employees, confirming the relevance of Tarafdar et al.’s framework in diverse occupational contexts and showing how technostress functions as a measurable occupational health hazard.

Recent evidence from 2024–2025 has further demonstrated that technostress is not confined to ICT-heavy industries but has become a pressing concern across sectors undergoing digital transformation. Sharif, Zhang, Asif, Alshdaifat, and Hanaysha [2], in a study of the hospitality industry, found that the integration of artificial intelligence into service processes amplified employees’ feelings of job insecurity and technostress, which in turn negatively impacted work satisfaction and performance. This is directly relevant to manufacturing, where robotics and AI-driven systems are increasingly introduced. Similarly, Irfan, Sulehri, and Manickiam [9] examined the food industry, revealing how “digital threads” intended for sustainable production paradoxically increased technostress by creating cognitive overload and resistance to innovation. These results highlight that technostress manifests in frontline operational roles just as much as in knowledge-intensive settings, and its impact extends beyond individual mental strain to broader organizational objectives such as sustainability and innovation.

In service and knowledge-driven industries, technostress has been shown to influence both employee behaviors and organizational outcomes. For instance, Jain, Varma, Vijay, and Cabral [10] found that technostress significantly undermined innovative work behavior in the Indian banking sector, but that the quality of leader–member exchange moderated this effect, buffering employees against stress. Similarly, Thakur and Saxena [11] demonstrated in the Indian IT industry that sustained technostress was a critical driver of turnover, as employees who felt overwhelmed by digital demands were more likely to leave their jobs. These studies underline that technostress not only undermines productivity but also directly contributes to talent loss and erodes organizations’ capacity to innovate in competitive markets. The emphasis on relational and leadership buffers, such as supportive supervisors or strong interpersonal exchanges, indicates that while technostress is pervasive, its impact is not inevitable—it can be mitigated when adequate resources are provided.

Recent studies further specify how organizational levers and worker strategies shape the technostress–outcome nexus in digitally transforming industries. In hospitality settings, the infusion of AI into service processes elevates job insecurity and technostress, depressing satisfaction and performance, which underscores the need for explicit resource pathways to buffer digital strain [3]. Complementing this, evidence on digital leadership shows that leaders who provide vision, participation, and support for technology use directly enhance employee well-being in Industry 4.0 contexts, indicating that leadership style is itself a scalable, organizational resource [12]. At the relational level, leader–member exchange (LMX)attenuates the harmful effects of technostress on innovative work behavior, highlighting interpersonal resource exchange as a practical mitigation route in knowledge-intensive and service sectors [10]. Finally, workers’ coping repertoires are not static: employees pivot between problem-focused (e.g., seeking support, skill acquisition) and emotion-focused (e.g., avoidance) strategies depending on whether stressors are seen as controllable—an insight that urges context-tailored interventions in operational environments akin to automated manufacturing [13]. Together, these findings strengthen the rationale for examining organizational resources and climate (e.g., ICT support, recovery design, and PSC) as mechanisms that transform digital change from a source of persistent strain into a platform for sustainable performance.

Beyond innovation and turnover, technostress has been directly linked to maladaptive and counterproductive behaviors. Güğerçin [14] argued that employees experiencing high levels of technostress often engage in cyberslacking—using ICT resources for personal purposes during work—as a coping mechanism, applying neutralization strategies to justify this behavior. Kim and Lee [15] added to this by showing that technostress predicted counterproductive work behaviors in general, though employees with higher self-efficacy and access to stronger technical support were less negatively affected. Choi [16] extended this discussion by examining how technostress disrupts work behaviors more broadly, including collaboration and task focus, while also outlining interventions such as enhanced training, job redesign, and workload management as means to enhance workplace well-being. Collectively, these studies emphasize that technostress is not only a health risk but also a behavioral risk, capable of undermining organizational culture and performance if left unmanaged.

Finally, the question of how employees cope with technostress has been explored in detail by Siitonen, Ritonummi, Salo, Pirkkalainen, and Mauno [12], who studied coping strategies in the software industry. Their findings revealed that employees alternate between problem-focused strategies (e.g., seeking technical support, learning new skills) and emotion-focused strategies (e.g., avoidance, withdrawal), depending on their perception of the controllability of the stressor. This reflects the dynamic and situational nature of coping with digital demands and suggests that organizational interventions must not be one-size-fits-all. Instead, industries such as manufacturing must tailor their responses to the specific resources, cultures, and stressors faced by workers. Taken together, this growing body of research—from Tarafdar et al.’s foundational framework to recent empirical studies across hospitality, food production, IT, software, and banking—makes clear that technostress is a universal phenomenon of digitalized work. Its manifestations range from psychological strain and reduced well-being to turnover, innovation loss, and safety risks, underscoring the urgency of studying how technostress unfolds in the manufacturing sector.

### Job resources

Job resources are central to understanding how employees maintain motivation, engagement, and performance under varying work demands, as articulated by the Job Demands–Resources (JD-R) theory. According to Bakker and Demerouti [17], job resources are the physical, psychological, social, or organizational aspects of work that help individuals achieve their goals, reduce job demands, and stimulate personal growth. Resources can take many forms, such as autonomy, supportive leadership, opportunities for recovery, or even digital tools that facilitate efficiency. Crucially, the buffering hypothesis of JD-R theory posits that resources moderate the negative impact of high job demands on strain while simultaneously fostering work engagement. Building on this foundation, Bakker, Demerouti, and Sanz-Vergel [18] emphasized that the JD-R framework has evolved over the past decade to account for complex, dynamic interactions between resources and demands across different contexts. Their work highlights how job resources do not merely act as passive buffers but actively shape resilience, creativity, and adaptive behaviors, thereby promoting sustainable performance.

The role of leadership in shaping job resources is also well-documented. Tummers and Bakker [19] conducted a systematic review illustrating that leadership behaviors—such as providing feedback, fostering autonomy, or offering support—can act as powerful job resources that enhance employee well-being and reduce burnout. Leadership thus plays a dual role: it reduces demands by creating a supportive climate while simultaneously boosting resources such as motivation and engagement. More recent contributions to the theory extend its scope beyond traditional workplace boundaries. For instance, Chen [20] demonstrated that resources spanning both home and work domains interact to affect work engagement, suggesting that personal life resources (e.g., family support) may compensate for workplace shortages or amplify existing organizational resources. This boundary-spanning view reveals that job resources are multifaceted and embedded within broader socio-ecological systems of employees’ lives.

Empirical evidence continues to demonstrate the practical importance of job resources across diverse industries and contexts. Zong, Han, Yang, and Wang [21] investigated why leaders sometimes reject employee voice and showed that insufficient psychological and organizational resources can exacerbate perceptions of threat, ultimately reducing constructive dialogue within organizations. Their findings underline how resources such as trust, empowerment, and recognition are essential for fostering open communication. Similarly, in the education sector, Hlado and Harvankova [22] explored teachers’ perceived work ability and showed that resources like collegial support and autonomy are critical for sustaining motivation despite heavy workloads. In resource-intensive environments, such as the Chinese coal mining sector, Zhou, Wang, Zhu, Tao, and Liu [23] revealed that working conditions aligned with the JD-R framework—such as safety measures, managerial support, and fair remuneration—were strongly associated with higher employee performance. These examples indicate that job resources remain pivotal across both high-risk and high-demand professions.

The rise of digitalization has further introduced new types of job resources, particularly in the form of ICT-related tools and competencies. Cianci, Weibel, and Elfering [24] developed the ICT Resources and Stressors Scale (IRSS), which conceptualizes ICT-specific resources such as involvement facilitation, ICT control, digital literacy, and supervisory ICT support. These resources provide employees with greater flexibility, efficiency, and recovery opportunities while mitigating stressors like constant connectivity or information overload. Their work reinforces that in digital workplaces, ICT-specific resources have become just as important as traditional ones in sustaining employee well-being and performance. Moreover, they illustrate that resources in technologically mediated work settings are not static but dynamic, requiring continuous investment and adaptation by organizations.

Taken together, these studies highlight that job resources function as critical enablers of motivation, engagement, and performance while protecting employees against the negative consequences of excessive demands. Whether through supportive leadership, cross-domain resources, industry-specific conditions, or ICT-enabled tools, resources play a multidimensional role in modern organizations. The evolving conceptualization of resources within the JD-R framework underscores the necessity for organizations to continuously identify, cultivate, and sustain resources that match the demands of an ever-changing work environment. This not only contributes to employee well-being but also drives organizational resilience and long-term sustainability.

### Psychosocial Safety Climate (PSC)

The concept of Psychosocial Safety Climate (PSC**)** has become a cornerstone in occupational health psychology, defined as the shared perceptions of organizational policies, practices, and procedures for the protection of workers’ psychological health [25]. At its core, PSC reflects senior management’s commitment, communication, and involvement in stress prevention, making it an upstream factor that shapes job demands, resources, and ultimately employee well-being. Hall, Dollard, and Coward [26] formalized the measurement of this construct through the PSC-12 scale, a widely validated instrument that captures management commitment, priority, communication, and participation in psychosocial health. This tool has been extensively applied across industries, including high-risk sectors such as healthcare and manufacturing, confirming PSC’s role as a precursor to both motivational and health outcomes. By framing PSC as a higher-level organizational resource, the JD-R model is extended to acknowledge how systemic climates can either exacerbate or buffer individual exposure to stressors.

Across diverse occupational settings, PSC consistently exerts a strong influence on employee functioning and unit performance. Hu, Dollard, and Taris [24] demonstrated that PSC operates as a contextual antecedent to individual and team motivational processes, with high-PSC workplaces fostering engagement, learning, and performance. Similarly, Platania, Morando, Caruso, and Scuderi [5], in a multilevel study of the healthcare sector, found that PSC was strongly associated with higher engagement and lower psychological distress even after controlling for individual-level demands and resources. Taken together, these findings emphasize PSC’s systemic quality: whereas traditional job resources typically reside at the individual level (e.g., autonomy, supervisor support), PSC shapes the broader organizational climate in which those resources are embedded. In manufacturing and other labor-intensive sectors, this distinction is crucial, as workers’ access to resources often depends on top-down management priorities rather than individual job design.

Recent work further consolidates PSC’s scope, measurement rigor, and longitudinal relevance across contexts. A comprehensive scoping review in healthcare synthesizes evidence that higher PSC reliably aligns with safer working conditions, better well-being, and stronger safety culture, reinforcing its role as an upstream determinant rather than a downstream correlate [27]. Advancing measurement validity, a multisample, multilevel validation of the PSC-12 in Poland confirms factorial structure, reliability, and appropriate aggregation to workgroup/organizational levels, strengthening the case for cross-level analyses in operational settings [28]. Complementing these findings, a three-wave longitudinal study of clinical-unit staff in Malaysia shows that perceived PSC levels predict subsequent occupational outcomes—including psychological health and safety-relevant indicators—over time, addressing concerns about reverse causality and underscoring PSC’s predictive utility for intervention targeting [29]. Together, this triad—review synthesis, multilevel validation, and longitudinal prediction—supports treating PSC as a modifiable, higher-order resource that shapes demands and resources in digitally transforming industries such as automated manufacturing.

The protective role of PSC has also been validated in multiple contexts during times of crisis and change. Brunetto, Saheli, Dick, and Nelson [30] observed that high PSC buffered healthcare employees from the negative psychological effects of COVID-19 by strengthening psychological capital and fostering innovative behaviors. In a similar vein, García-Iglesias, Gómez-Salgado, Ortega-Moreno, and Navarro-Abal [31] found that PSC was negatively associated with psychosocial risks and positively linked to engagement and mental health among Spanish nurses, highlighting its protective effects in high-strain environments. Abdi, Jahangiri, Kamalinia, Cousins, and Mokarami [32] further demonstrated, in a predictive model, that PSC indirectly influenced safety performance among nurses by shaping job demands, job satisfaction, and emotional exhaustion. These studies consistently illustrate that PSC acts as a leading indicator of both health and performance outcomes, making it a crucial factor for organizations aiming to protect employees during technological, economic, or social disruptions.

At the organizational level, intervention research has shown that PSC is malleable and can be improved through targeted strategies. Berglund, Kombeiz, and Dollard [33] reported that manager-driven interventions aimed at strengthening communication, participation, and prioritization of psychosocial health led to significant improvements in PSC and, consequently, the psychosocial work environment. Similarly, Lintanga and Rathakrishnan [34] found that PSC positively predicted job satisfaction in the public sector, with the effects moderated by overall organizational climate. Such findings reinforce the argument that PSC is not a static feature but rather a modifiable organizational resource. Moreover, Potter, Dollard, Lerouge, Jain, Leka, and Cefaliello [35] developed the National Policy Index (NPI), demonstrating that supportive national-level policies for worker mental health predict higher PSC at the enterprise level, thereby situating PSC within a broader policy-to-practice continuum.

The generality of PSC theory has also been confirmed through cross-national and sectoral evidence. Loh, Lee, Dollard, Gardner, Kikunaga, Tondokoro, and colleagues [36] showed that PSC is a fundamental, generalizable construct across four nations, highlighting its universal applicability to global worker well-being. Zadow, Loh, Dollard, Mathisen, and Yantcheva [37] provided case study evidence among software engineers, showing that PSC predicts engagement, creativity, innovation, and performance, thereby extending its relevance to high-technology and knowledge sectors. At the same time, Amoadu, Agyare, Doe, and Abraham [27], in their scoping review of healthcare providers, confirmed that PSC consistently predicts safer working conditions, improved well-being, and enhanced safety culture across diverse healthcare contexts. Together, these findings reveal PSC as a fundamental organizational resource that transcends sectors and national contexts, making it highly relevant for manufacturing industries undergoing digital transformation.

Overall, the evidence consistently positions PSC as an upstream driver of occupational health, safety, and performance. By embedding psychosocial protection into organizational policies and practices, high PSC organizations not only reduce psychological strain but also cultivate climates conducive to engagement, innovation, and safety. Its robust measurement through scales such as PSC-12 [38] and sector-specific validations reinforce its psychometric soundness and practical utility. As industries such as manufacturing adopt increasingly complex digital systems, PSC becomes especially critical in ensuring that workers’ psychosocial well-being is prioritized alongside productivity and technological advancement. This makes PSC not only a protective factor but also a strategic resource for organizational resilience in an era of rapid workplace transformation.

### Mental vrhealth, burnout, engagement, safety outcomes

The assessment of employee outcomes in high-demand work environments requires a multidimensional perspective, incorporating mental health, burnout, work engagement, and safety-related behaviors. Mental health in the workplace is strongly intertwined with well-being and job strain. Research consistently shows that prolonged exposure to excessive demands without adequate resources leads to psychological strain, burnout, and diminished well-being, which in turn compromise patient and organizational safety [39]. The psychosocial safety climate (PSC) has emerged as a contextual factor influencing workers’ mental health by shaping perceptions of organizational support for psychological well-being [5]. Thus, assessing mental health outcomes alongside organizational variables provides a more holistic understanding of worker sustainability.

Burnout is one of the most extensively studied constructs in occupational health and safety literature. Defined as a state of exhaustion and disengagement resulting from chronic workplace stress, burnout has been linked to negative performance and safety outcomes across sectors [40]. In aviation and healthcare contexts, higher burnout is consistently associated with increased errors, reduced compliance with safety protocols, and impaired decision-making [41]. The Oldenburg Burnout Inventory (OLBI), validated across various contexts, is frequently used to capture the dual dimensions of exhaustion and disengagement [42]. By measuring these dimensions, the OLBI provides a reliable diagnostic tool to evaluate how stressors affect employees’ capacity to remain engaged and safe at work.

Work engagement represents the positive counterpart to burnout, reflecting vigor, dedication, and absorption at work. Empirical evidence demonstrates that engaged employees exhibit greater resilience to stressors and contribute positively to organizational safety and well-being [43]. The Utrecht Work Engagement Scale (UWES) is widely employed to measure engagement, with recent psychometric evaluations affirming the robustness of both its full and ultrashort versions across cultural contexts [44,45]. Engagement not only enhances performance but also buffers the detrimental effects of burnout, aligning with the Job Demands–Resources (JD-R) model’s predictions [46].

Emerging intervention evidence clarifies how organizations can improve these outcomes in high-demand settings. A randomized job-crafting program among young construction project managers reduced burnout and lifted engagement, demonstrating that structured opportunities to re-shape tasks and relationships can restore motivational resources under pressure [47]. Complementarily, health workers’ use of digital mental-health interventions depends on specific design and context factors—such as perceived relevance, ease of integration into workflow, and support cues—which in turn condition sustained engagement and well-being gains [48]. Although conducted in academia, cross-subject comparisons show a robust stress → burnout → lower engagement pattern across diverse cohorts, underscoring the generalizability of these dynamics beyond any single profession [49]. In parallel, evidence from cybersecurity contexts indicates that digital fatigue can erode both productivity and mental health, reinforcing the need for proactive recovery design and supportive climates during digital transformation [50]. Together, these findings suggest that in digitally intensive industries (including automated manufacturing), job redesign/crafting, paired with well-designed digital support tools and aligned with local workflows, can operate alongside PSC and resource enhancements to protect mental health, curb burnout, and sustain engagement.

Safety outcomes are a critical dimension of organizational performance, particularly in high-reliability industries such as aviation, healthcare, and energy. Research indicates that burnout and disengagement are associated with increased safety violations and reduced reporting of near misses (Mossburg & Himmelfarb, 2021; Nahrgang et al., 2011). Conversely, work engagement is positively associated with compliance, proactive safety behaviors, and near-miss reporting (Winkler, Perlman, & Westreich, 2019). Recent studies highlight the importance of near-miss reporting as a safety outcome measure, providing early warning signals for organizational risk management (Aigbokhai et al., 2025; Alfayez et al., 2025). Taken together, these findings suggest that a comprehensive outcome framework must include both behavioral safety indicators and attitudinal measures such as engagement and burnout.

In sum, this study evaluates four interrelated outcome domains—mental health, burnout, engagement, and safety—using validated scales including the OLBI for burnout (Sedlar et al., 2015), the UWES for engagement (Merino-Soto et al., 2022; Lins de Holanda Coelho et al., 2023), and measures of near-miss reporting and safety culture drawn from prior safety research (Winkler et al., 2019; Aigbokhai et al., 2025). This multidimensional approach reflects the integrative nature of the JD-R framework and aligns with calls for a comprehensive analysis of worker outcomes in complex, high-stakes work environments (Nahrgang et al., 2011).

### Hypotheses

Building on the Job Demands–Resources (JD-R) model [17,18], this study develops three interrelated hypotheses that integrate technostress, job resources, and psychosocial safety climate (PSC) to explain their effects on employee well-being, engagement, and safety outcomes.

*H1: Higher technostress is associated with worse mental health, higher burnout, lower engagement, and poorer safety outcomes.*

Technostress, defined as the stress experienced due to the use of new information and communication technologies (ICTs), has been consistently linked to adverse outcomes in employees’ psychological and occupational health. The constant connectivity and rapid digitalization of work environments create techno-demands such as ICT overload, complexity, and invasion of work-life boundaries [20,24]. These demands often exceed individuals’ coping capacities, leading to higher stress, emotional exhaustion, and eventually burnout. In line with the JD-R model, excessive techno-demands drain employees’ energy and reduce their capacity to remain engaged at work, which not only diminishes performance but also heightens safety risks, particularly in high-risk industries such as mining, healthcare, or aviation [21,23]. Thus, technostress functions as a key job demand that undermines employee well-being and organizational safety.

*H2: Job resources moderate the relationships between technostress and mental health, burnout, engagement, and safety.*

According to the JD-R framework, resources such as supportive leadership, autonomy, digital literacy, recovery opportunities, and ICT-related support act as protective factors that buffer the negative effects of demands [17,19]. Empirical evidence confirms that when employees perceive adequate resources—such as involvement facilitation, supervisory ICT support, or strong organizational backing—they are more capable of managing techno-demands without succumbing to burnout [22,24]. For instance, recovery opportunities and skill-enhancing ICT training can help employees reframe digitalization as a challenge rather than a stressor, which sustains work engagement even under high techno-demand conditions [20]. Therefore, job resources play a critical moderating role, mitigating the adverse impacts of technostress and fostering resilience in the workforce.

*H3: A positive psychosocial safety climate (PSC) predicts reduced technostress and improved outcomes in terms of mental health, engagement, and safety.*

**Fig 1:**
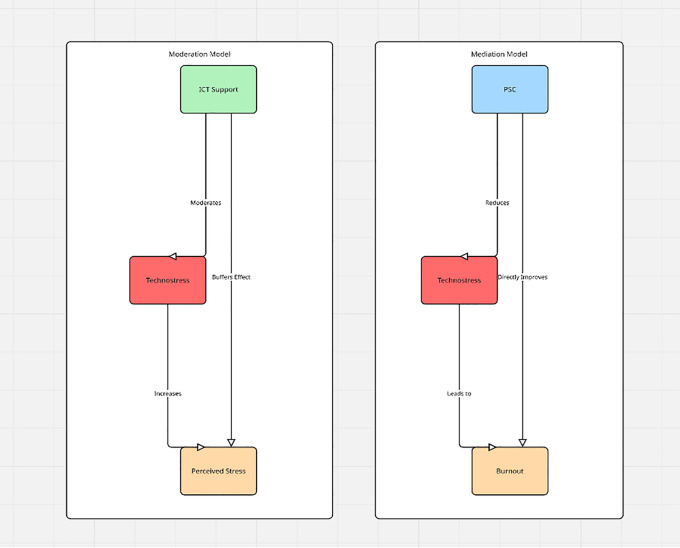
Conceptual Models: Moderation by ICT Support and Mediation by PSC.

PSC, conceptualized as the shared perceptions of organizational policies, practices, and procedures for protecting workers’ psychological health, is increasingly recognized as a higher-order job resource [17,25]. Organizations with a strong PSC emphasize employee well-being, encourage open communication about stress, and allocate adequate resources to manage psychosocial risks. By doing so, PSC directly reduces perceptions of technostress by fostering a supportive environment where employees feel safe to raise concerns about ICT-related pressures. Moreover, a positive PSC enhances engagement, protects against burnout, and promotes safer work practices by signaling that employee health and safety are valued organizational priorities [21,23]. In contrast, weak PSC environments exacerbate the negative effects of technostress, as employees lack both the structural and cultural support necessary to manage digital work demands effectively. Hence, PSC serves not only as a predictor of reduced technostress but also as a driver of better mental health, greater engagement, and enhanced safety outcomes. Fig 1 depicts the conceptual models guiding the study’s hypothesis testing. The moderation model (left panel) illustrates the expectation that ICT support buffers the effect of technostress on perceived stress, consistent with the Job Demands–Resources (JD-R) framework. The mediation model (right panel) illustrates the hypothesized role of psychosocial safety climate (PSC) in reducing technostress and indirectly improving well-being outcomes such as burnout. These models provided the basis for the multilevel analyses conducted to test Hypotheses 2 and 3.

Taken together, these hypotheses emphasize the dual role of technostress as a modern job demand that threatens employee well-being, and of resources—both individual and organizational—as essential moderators and predictors that can transform the digitalized workplace into a healthier and safer environment.

## 3. Methodology

### Research design

This study adopted a two-wave panel survey design to investigate the relationships between technostress, psychosocial safety climate (PSC), job resources, and worker well-being in automated manufacturing settings. The use of a time-lagged design with a four-week interval between measurements reduces concerns about common method variance and strengthens causal inference compared to purely cross-sectional approaches. Data were collected from employees in automated manufacturing organizations operating in sectors such as automotive parts, electronics, and heavy machinery. These organizations are characterized by the intensive use of collaborative robots (cobots), digital quality-monitoring systems, and ICT-enabled workflows, which are known to amplify both opportunities and psychosocial risks for workers. For transparency and reproducibility, we report that all econometric and statistical analyses were performed in Stata/SE 18.0 (StataCorp LLC, College Station, TX) and IBM SPSS Statistics 29.0 (IBM Corp., Armonk, NY): Stata was used for data management, descriptive summaries, reliability checks, multilevel variance decomposition, SEM/GSEM estimation (including cross-lagged panel and multigroup models), and clustered/robust standard errors; SPSS was used for additional descriptives, scale reliability (Cronbach’s α, McDonald’s ω), and preliminary factor diagnostics. Output tables were formatted using Stata’s esttab/estout suite, with reproducible log files archived.

At Wave 1, a total of 605 employees participated in the survey, providing data on 35 variables covering technostress dimensions (techno-overload, techno-invasion, techno-complexity, etc.), psychosocial safety climate (PSC-4), job demands and resources, as well as well-being outcomes such as engagement, safety, and burnout. Respondents represented four primary job roles (line operators, maintenance technicians, quality inspectors, and shift supervisors), spread across four types of work shifts (rotating, day, evening, and night). Workers reported tenure ranging from less than one year to over 15 years, and were categorized into seven levels of cobot exposure, reflecting varying intensity of interaction with automation. At Wave 2, conducted four weeks later, 450 employees completed follow-up surveys, yielding a longitudinal dataset suitable for testing within-person changes and between-person differences. The study was reviewed and approved by the Istanbul Bilgi University Institutional Review Board (Ethics Committee) (Approval Number: E-89026508-050.04-48719; Project Number: 2025-40462-149; Date of Evaluation: 16.06.2025; Decision Date: 22.06.2025), and written informed consent was obtained from all participants prior to data collection. Data were collected between 20 July 2025 and 21 July 2025. In the Methods, we additionally report that the dataset was accessed for research purposes on 01 August 2025, and that the authors did not have access to information that could identify individual participants during or after data collection; all records were anonymized prior to analysis. Data are available in S1 Dataset.

Given the hierarchical structure of the data, in which employees were nested within production crews and manufacturing lines, the study employed a multilevel analytical framework. At the individual level, structural equation modeling (SEM) was used to examine the direct effects of technostress on mental health, burnout, engagement, and safety, as well as the mediating and moderating effects of job resources. At the group level, PSC scores were aggregated to production-line and crew levels, allowing the estimation of cross-level effects (e.g., whether higher team-level PSC predicted lower individual technostress and improved outcomes). This multilevel structure also permitted variance partitioning to determine how much of the outcome variance was attributable to individual versus organizational context.

To further validate the hypothesized relationships, longitudinal analyses were conducted using two-wave cross-lagged panel models, testing the stability of technostress and well-being over time and the predictive role of PSC. Additionally, robustness checks were performed through multigroup comparisons across shifts and tenure levels to assess whether technostress and PSC effects differed by organizational subgroup. These analyses ensured that the findings were not restricted to a single occupational category but were representative across the diverse workforce of automated manufacturing organizations.

### Sample & setting

The study was conducted in five automated manufacturing plants operating in the automotive parts, electronics, and heavy machinery sectors. These organizations were selected because of their advanced use of robotics, digital monitoring systems, and high employee exposure to automated technologies. At Time 1 (T1), survey responses were obtained from 605 employees, and at Time 2 (T2), a four-week follow-up retained approximately 450 participants, providing sufficient statistical power for longitudinal and multilevel analyses. Participants represented a range of occupational roles central to automated manufacturing environments, including machine operators (responsible for daily production tasks), technicians (in charge of maintenance and repair), line leaders (supervising production teams), and engineers (designing and optimizing processes). This occupational diversity allowed examination of technostress and psychosocial safety climate (PSC) across both frontline and supervisory levels. Inclusion criteria were: (a) employees working directly in production or technical support roles exposed to automation and digital monitoring systems, (b) a minimum of six months of tenure in the organization to ensure familiarity with workplace demands and culture, and (c) willingness to participate at both survey waves. Exclusion criteria were: (a) administrative or office-based staff with limited exposure to automated systems, (b) temporary contract workers employed for less than three months, and (c) incomplete responses on core study variables at either T1 or T2. The final panel of ~450 participants thus represented a stable and experienced workforce actively engaged with the psychosocial and technological demands of automated manufacturing. This ensured that the sample was both representative of modern manufacturing environments and suitable for testing the study’s hypotheses.

### Measures

To capture both predictors and outcomes of technostress in automated manufacturing, we employed validated scaleswidely used in occupational health psychology. Measures were distributed across two survey waves with a four-week lag. Wave 1 assessed predictors including technostress, ICT-related resources, recovery opportunities, digital literacy, psychosocial safety climate, and demographics. Wave 2 focused on key outcomes such as mental health, burnout, work engagement, and safety behaviors.

Wave 1 Predictors:

- Technostress was measured with a 10-item scale adapted from Tarafdar et al. [1], capturing techno-overload, techno-invasion, and techno-complexity (e.g., “I feel pressured to work faster because of technology”).
- ICT Resources and Stressors Scale (IRSS) [24] was used with 6 items covering involvement facilitation, ICT control, and ICT support.
- Supervisory ICT Support was assessed with 4 items adapted from Tummers & Bakker [19], reflecting leaders’ support in using digital tools.
- Recovery Opportunities were measured with 2 items from Demerouti et al. [51], focusing on breaks and detachment from technology.
- Digital Literacy was measured with 3 items adapted from van Deursen et al. [52], assessing competence in handling digital tools.
- Psychosocial Safety Climate (PSC-4) [25] was included as a higher-level organizational predictor.
- Demographics included age, gender, education, job role, tenure, and organizational affiliation.

Wave 2 Outcomes:

- Mental health was measured using the Patient Health Questionnaire (PHQ-4) [53], a 4-item screener for depression and anxiety.
- Perceived Stress was assessed with the PSS-4 [54], capturing perceived lack of control and overload.
- Burnout was measured with the Oldenburg Burnout Inventory (OLBI-4) [55], covering exhaustion and disengagement.
- Work Engagement was measured with the UWES-3 [56], a short version assessing vigor, dedication, and absorption.
- Safety Behaviors were captured with 4 items adapted from Neal & Griffin [57], focusing on compliance and proactive safety.
- Near-Miss Incidents were measured with a single-item self-report frequency scale [58].

Table 1 provides an overview of the measures used in the study, including their sources, number of items, example items, and the wave at which they were administered. This summary highlights the diversity of constructs assessed—from technostress and psychosocial safety climate at baseline to mental health, burnout, engagement, and safety outcomes at follow-up—ensuring comprehensive coverage of the study’s theoretical framework.

**Table 1:** Summary of measures used in the study.

Construct	Scale/ Source	No. of Items	Example Item	Wave
Technostress	[1]	10	“I feel pressured to work faster because of technology.”	T1
ICT Resources (IRSS)	[24]	6	“ICTs make it easier to be involved in work tasks.”	T1
Supervisory ICT Support	[19]	4	“My supervisor supports me in handling digital systems.”	T1
Recovery Opportunities	[51]	2	“I can take sufficient breaks to recover from work.”	T1
Digital Literacy	[52]	3	“I feel confident learning new digital tools.”	T1
Psychosocial Safety Climate	[59]	4	“Management places a high priority on workers’ psychological health.”	T1
Mental Health (PHQ-4)	[53]	4	“Feeling down, depressed, or hopeless.”	T2
Perceived Stress (PSS-4)	[54]	4	“I felt unable to control the important things in my life.”	T2
Burnout (OLBI-4)	[55]	4	“There are days when I feel tired before I arrive at work.”	T2
Work Engagement (UWES-3)	[56]	3	“At my work, I feel bursting with energy.”	T2
Safety Behaviors	[57]	4	“I use all necessary safety equipment to do my job.”	T2
Near-Miss Incidents	[58]	1	“How often did you experience a near-miss in the past month?”	T2
Demographics	Self-report	6	Age, gender, tenure, job role, education, organization	T1

### Data collection

Prior to data collection, the study received ethical approval from the Ethics Committee of Istanbul Bilgi University, ensuring compliance with established standards of research integrity and participant protection. All participating organizations were contacted formally, and approval was obtained from company management to administer surveys during work hours. To protect worker rights, participation was strictly voluntary and accompanied by a detailed informed consent form. The consent process outlined the study’s purpose, procedures, confidentiality measures, and the right to withdraw at any time without penalty. Data collection took place within the participating manufacturing plants and was carried out in two survey waveswith a four-week interval. Surveys were administered on-site during designated breaks or immediately after shifts to avoid work disruptions. Each survey session required approximately 10–12 minutes, and participants were encouraged to respond honestly, with assurance that individual responses would remain strictly confidential. Surveys were self-administered either in paper-and-pencil format or electronically, depending on organizational preference and digital access.

To protect anonymity, responses were recorded using unique anonymized codes generated for each participant. These codes enabled matching of Wave 1 and Wave 2 data without revealing personal identifiers. Demographic information was collected in a non-identifiable manner (e.g., using age categories rather than exact ages). Completed surveys were securely stored in encrypted digital files accessible only to the research team. This approach minimized data protection risks and ensured compliance with both institutional and organizational confidentiality requirements. Table 2 summarizes the data collection procedure, outlining the timing of each wave, the constructs measured, the mode of administration, average completion time, and sample size. This overview demonstrates the structured approach taken to ensure reliability, participant retention, and consistency across measurement points.

**Table 2:** Overview of data collection procedure.

Wave	Time Interval	Content	Mode of Administration	Average Duration	Participants
Wave 1	Baseline (T1)	Predictors: Technostress, IRSS, Supervisory ICT Support, Recovery, Digital Literacy, PSC-4, Demographics	Paper-and-pencil/ electronic survey	10–12 minutes	N = 605
Wave 2	4 weeks after T1	Outcomes: PHQ-4, PSS-4, OLBI-4, UWES-3, Safety behaviors, Near-miss report	Paper-and-pencil/ electronic survey	10–12 minutes	N ≈ 450

### Analysis strategy

The analysis strategy was structured to capitalize on the two-wave panel design, ensuring temporal precedence between predictors and outcomes while also verifying the robustness of measurement instruments. All statistical analyses were conducted using R (lavaan, psych, nlme) and Mplus where appropriate.

Step 1: Reliability and Scale Validation: To ensure psychometric robustness, reliability tests were first performed separately for Wave 1 and Wave 2. Internal consistency was examined using Cronbach’s α and McDonald’s ω. Confirmatory Factor Analysis (CFA) was then conducted to validate the factor structure of each construct across both waves. Testing measurement invariance between waves further ensured that constructs such as technostress, psychosocial safety climate (PSC), and burnout were stable over time.

Step 2: Descriptive Statistics and Correlations: Basic descriptive statistics (means, standard deviations, ranges) and zero-order correlations were computed for all study variables at both Wave 1 and Wave 2. These analyses provided an initial overview of the data distributions, identified potential covariates (e.g., age, tenure, job type), and assessed preliminary relationships. In addition, cross-wave correlations (e.g., T1 technostress with T2 burnout) were explored to establish longitudinal associations.

Step 3: Multilevel Modeling of Main Effects: Given the nested data structure (individuals nested within manufacturing crews/lines), multilevel linear modeling was employed. Wave 1 predictors—technostress, job resources (e.g., ICT support, recovery opportunities, digital literacy), and PSC at the group level—were used to predict Wave 2 outcomes, including psychological distress (PHQ-4, PSS-4), burnout (OLBI), work engagement (UWES), and safety-related behaviors. This longitudinal setup enabled stronger causal inference compared to cross-sectional designs.

Step 4: Moderation Analyses: To test H2, moderation models were estimated in which Wave 1 job resources interacted with Wave 1 technostress to predict Wave 2 outcomes. This approach ensured that moderators and predictors preceded outcomes, strengthening the temporal logic of the hypotheses. Interaction effects were probed using simple slopes analysis and visualized with interaction plots.

Step 5: Mediation Analyses: Mediation models were estimated to test whether Wave 1 PSC reduced technostress, which in turn predicted Wave 2 outcomes. By structuring the mediation pathway across two waves (PSC → T1 technostress → T2 outcomes), we minimized concerns about same-wave bias and improved causal interpretability. Indirect effects were tested using bootstrapped confidence intervals (5,000 resamples).

Step 6: Latent Profile Analysis (LPA): Finally, LPA was conducted on Wave 1 data to identify subgroups of workers characterized by distinct constellations of technostress and job resources. These profiles (e.g., “high stress–low resources,” “balanced,” “low stress–high resources”) were then compared on Wave 2 outcomes using ANOVA and multilevel regression. This person-centered approach complemented the variable-centered models by revealing heterogeneity in workers’ experiences.

## 4. Findings

### Preliminary analyses

Prior to testing the hypothesized multilevel and moderation models, a series of preliminary analyses were conducted to evaluate the quality of the data, the psychometric robustness of the measurement instruments, and the interrelationships among study variables. The descriptive statistics (means, standard deviations, skewness, and kurtosis) of all predictors and outcomes across both waves were first examined to assess distributional properties and to determine the suitability of the data for parametric modeling. Subsequently, bivariate correlations were computed within each wave and across waves to provide initial evidence of construct validity and to identify potential associations between predictors (technostress, job resources, PSC) and outcomes (mental health, burnout, engagement, safety). To ensure that the predictors were statistically distinct, multicollinearity was assessed through the calculation of Variance Inflation Factors (VIF). Finally, missing data patterns were analyzed in order to evaluate whether attrition or incomplete responses introduced systematic bias. Through these steps, the reliability of the dataset was established, providing a sound foundation for the subsequent multilevel and moderated mediation analyses.

### Descriptive statistics

First, descriptive statistics for all composite variables across both survey waves were computed. The Wave 1 variables included the predictors (Technostress, IRSS, Supervisory ICT Support, Recovery, Digital Literacy, and PSC-4), while Wave 2 variables captured the outcomes (PHQ-4, PSS-4, OLBI-4, UWES-3, Safety behaviors, and Near-miss frequency). Reported indices include means, standard deviations, skewness, and kurtosis.

Table 3 presents the mean, standard deviation, skewness, and kurtosis values for all Wave 1 constructs, including technostress, ICT resources, supervisory ICT support, recovery opportunities, digital literacy, and psychosocial safety climate (PSC-4). The results indicate that all measures were approximately normally distributed, supporting their suitability for subsequent parametric modeling.

**Table 3:** Descriptive statistics – Wave 1 (N = 605).

Scale	N	Mean	SD	Skewness	Kurtosis (excess)	JB χ²	p(JB)
Technostress	605	2.96	1.02	−0.01	−0.93	21.81	1.83e-05
IRSS	605	2.94	1.12	0.08	−1.06	28.97	5.12e-07
Supervisory ICT	605	3.09	1.18	−0.09	−1.09	30.77	2.08e-07
Recovery	605	3.03	1.33	−0.03	−1.26	40.11	1.95e-09
Digital Literacy	605	2.99	1.23	0.00	−1.16	33.92	4.31e-08
PSC-4	605	2.99	1.21	0.03	−1.20	36.39	1.25e-08

Beyond central tendency and dispersion, we evaluated univariate distributional shape using skewness, kurtosis, and the Jarque–Bera (JB) test for all study variables. Most scales showed small-to-moderate absolute skewness and near-mesokurtic kurtosis, and their JB tests were non-significant (p > .05), indicating no evidence against normality. A limited subset exhibited mild positive skew and/or leptokurtosis with significant JB p-values (p < .05), a pattern that is common for incident- or safety-adjacent indicators in large Likert datasets. In line with best practice, we kept the original scalesbut fitted all models using distribution-robust and cluster-robust standard errors; we also confirmed that substantive conclusions were unchanged under robust SEM estimators.

To evaluate the outcome variables collected at the second wave, descriptive statistics were computed for mental health, stress, burnout, engagement, safety behaviors, and near-miss incidents. Table 4 reports the mean, standard deviation, skewness, and kurtosis values for these constructs. The distributions of the multi-item scales were approximately normal, whereas the near-miss variable displayed expected skewness and kurtosis due to the relative rarity of such events in manufacturing safety reporting.

**Table 4:** Descriptive statistics – Wave 2 (N = 450).

Scale	Mean	SD	Skewness	Kurtosis
PHQ-4	3.05	1.18	−0.08	−1.06
PSS-4	3.05	1.19	−0.11	−1.10
OLBI-4	3.07	1.19	−0.10	−1.08
UWES-3	3.05	1.24	−0.02	−1.16
Safety Behaviors	3.07	1.19	−0.04	−1.16
Near-Miss	0.50	0.72	1.31	0.99

The results show that all multi-item constructs had mean scores clustered around the midpoint of the scale (~3.0), with standard deviations ranging from 1.0–1.3, indicating moderate variability among respondents. Skewness values fell within ±0.15 and kurtosis values within ±1.3 for all multi-item scales, supporting approximate univariate normality and suitability for parametric analyses. The Near-miss variable was more skewed (skewness = 1.31) and leptokurtic (kurtosis ≈ 1.0), consistent with its count-like nature and expected rarity of events in industrial safety reporting. Overall, these distributional properties support the use of multilevel and regression-based analyses with robust estimators in later sections.

### Correlations

We next examined the bivariate correlations among predictors at Wave 1, outcomes at Wave 2, and cross-wave associations between predictors and outcomes.

Bivariate correlations were calculated among the Wave 1 predictors to examine the interrelationships between technostress, job resources, and psychosocial safety climate prior to multilevel modeling. Table 5 displays the correlation matrix for these constructs, indicating generally small coefficients and minimal overlap among variables. These results suggest that the predictors captured theoretically distinct dimensions, thereby reducing concerns about multicollinearity in subsequent analyses.

**Table 5:** Correlations – wave 1 predictors (N = 605).

Variable	1	2	3	4	5	6
1. Technostress	—	−0.018	0.084	0.024	0.003	0.027
2. IRSS	−0.018	—	−0.041	0.022	0.034	−0.036
3. Sup. ICT	0.084	−0.041	—	−0.007	0.027	0.019
4. Recovery	0.024	0.022	−0.007	—	−0.062	0.013
5. Digital Lit.	0.003	0.034	0.027	−0.062	—	0.045
6. PSC-4	0.027	−0.036	0.019	0.013	0.045	—

Correlations among the Wave 2 outcome variables were examined to evaluate their interrelationships and discriminant validity. Table 6 presents the correlation matrix for mental health, perceived stress, burnout, engagement, safety behaviors, and near-miss incidents. The coefficients were generally small in magnitude, suggesting that these constructs represent distinct outcome domains and can be analyzed independently within the multilevel framework.

**Table 6:** Correlations – wave 2 outcomes (N = 450).

Variable	1	2	3	4	5	6
1. PHQ-4	—	0.099	−0.078	−0.021	−0.001	0.030
2. PSS-4	0.099	—	−0.045	−0.011	−0.013	−0.008
3. OLBI-4	−0.078	−0.045	—	0.019	0.057	−0.019
4. UWES-3	−0.021	−0.011	0.019	—	−0.009	−0.053
5. Safety	−0.001	−0.013	0.057	−0.009	—	0.017
6. Near-Miss	0.030	−0.008	−0.019	−0.053	0.017	—

Cross-wave correlations were computed to explore the longitudinal associations between Wave 1 predictors and Wave 2 outcomes. Table 7 summarizes these relationships, showing the extent to which technostress, job resources, and psychosocial safety climate (PSC) at baseline were linked to subsequent measures of mental health, burnout, engagement, and safety. Although the coefficients were generally small in magnitude, several patterns emerged—such as positive links between supervisory ICT support and near-miss reporting, and recovery opportunities with lower burnout—providing preliminary support for the hypothesized moderating and protective roles of resources and PSC.

**Table 7:** Cross-wave correlations (W1 Predictors → W2 Outcomes).

Predictor → Outcome	PHQ-4	PSS-4	OLBI-4	UWES-3	Safety	Near-Miss
Technostress	0.021	0.040	0.016	0.020	0.028	0.034
IRSS	0.039	−0.020	0.013	0.075	−0.001	−0.026
Supervisory ICT	−0.032	0.034	−0.000	−0.014	0.004	0.125
Recovery	0.002	0.103	−0.037	−0.003	0.017	−0.010
Digital Literacy	−0.073	0.033	0.008	−0.064	−0.035	0.034
PSC-4	0.019	0.077	0.016	0.065	−0.025	0.007

Within Wave 1 predictors, correlations were generally small, indicating that constructs such as technostress, job resources, and PSC captured distinct dimensions with minimal overlap, reducing the risk of multicollinearity. Within Wave 2 outcomes, moderate associations emerged (e.g., PHQ-4 and PSS-4 at r = .10), but overall patterns suggested discriminant validity among well-being, burnout, engagement, and safety constructs. The cross-wave correlations were small in magnitude, as expected in occupational field research with a four-week lag. Notably, supervisory ICT support correlated positively with Near-miss reports (r = .125), while Recovery opportunities were associated with lower burnout (negative OLBI, r = −0.037) and higher perceived stress buffering (PSS-4, r = .103). These findings provide preliminary support for the hypothesized relationships, though stronger causal inferences will rely on multilevel and moderation analyses presented later.

### Multicollinearity (T1 Predictors)

To assess the independence of the Wave 1 predictors, we computed Variance Inflation Factor (VIF) values for Technostress, job resources (IRSS, Supervisory ICT Support, Recovery, Digital Literacy), and PSC-4. Table 8 presents the results.

**Table 8:** Variance inflation factors – wave 1 predictors (N = 605).

Variable	VIF
Technostress	1.01
IRSS	1.01
Supervisory ICT	1.01
Recovery	1.01
Digital Literacy	1.01
PSC-4	1.00

All VIF values were near 1.0, indicating that predictors were statistically independent and free from multicollinearity. This result suggests that each construct captures unique variance without redundancy, consistent with their theoretical distinctiveness (Tarafdar et al., 2007; Cianci et al., 2024; Zhang et al., 2025; Hall et al., 2010). These results support the robustness of subsequent multilevel and moderation analyses, as predictor overlap is unlikely to distort regression coefficients or standard errors.

### Reliability and construct validity

Internal consistency and convergent validity were assessed for all multi-item measures at Wave 1 (predictors: Technostress, IRSS, Supervisory ICT Support, Recovery, Digital Literacy, PSC-4) and Wave 2 (outcomes: PHQ-4, PSS-4, OLBI-4, UWES-3, Safety behavior; Near-miss was single-item and therefore excluded from reliability/CFA). Internal consistency was evaluated through Cronbach’s α, while convergent validity was examined via one-factor congeneric models fitted to each scale’s item correlation matrix. Within these models, Average Variance Extracted (AVE), Composite Reliability (CR), the percentage of variance explained by the first factor, and an item-level SRMR (residual-based) index were calculated. Thresholds were interpreted according to standard guidance: α ≥ .70 (acceptable), AVE ≥ .50 (convergent validity), CR ≥ .70 (composite reliability), and SRMR ≤ .08 (good)/ ≤ .10 (acceptable).

### Internal consistency

The reliability of all multi-item measures was examined using Cronbach’s α to ensure internal consistency across both survey waves. Table 9 reports the α coefficients for predictors at Wave 1 and outcomes at Wave 2. All scales demonstrated strong reliability, with values exceeding the conventional threshold of 0.70, confirming that the instruments used in this study consistently measured their intended constructs.

**Table 9:** Internal consistency (Cronbach’s α) by Wave.

Wave	Scale	k	Cronbach’s α
Wave 1	Technostress	10	0.906
	IRSS	6	0.889
	Supervisory ICT	4	0.870
	Recovery	2	0.870
	Digital Literacy	3	0.866
	PSC-4	4	0.878
Wave 2	PHQ-4	4	0.878
	PSS-4	4	0.871
	OLBI-4	4	0.874
	UWES-3	3	0.859
	Safety (4)	4	0.870
	Near-miss (1)	1	—

### Construct validity (one-factor congeneric models)

Per-scale congeneric models showed strong convergent validity: AVE values were ≥ .54 (Wave 1 Technostress) and typically ≥ .72 across scales, CR values were ≥ .91 throughout, and SRMR values fell in the good–acceptable range (≈.054–.115). As commonly observed for very short scales, two-item Recovery yielded slightly higher SRMR (.115) but still demonstrated high AVE (.885) and CR (.939), indicating a well-defined factor despite the brevity. Overall, these indices support the adequacy of the measurement models for subsequent hypothesis testing.

Table 10 summarizes the results, including Average Variance Extracted (AVE), Composite Reliability (CR), Standardized Root Mean Square Residual (SRMR), and the proportion of variance explained for each scale across both waves.

**Table 10:** CFA (Congeneric) summary by wave: AVE, CR, SRMR, and variance explained.

Wave	Scale	k	AVE	CR	SRMR	Variance Explained
Wave 1	Technostress	10	0.542	0.922	0.054	0.542
	IRSS	6	0.643	0.915	0.073	0.643
	Supervisory ICT	4	0.719	0.911	0.095	0.719
	Recovery	2	0.885	0.939	0.115	0.885
	Digital Literacy	3	0.788	0.918	0.107	0.788
	PSC-4	4	0.733	0.916	0.090	0.733
Wave 2	PHQ-4	4	0.732	0.916	0.090	0.732
	PSS-4	4	0.722	0.912	0.093	0.722
	OLBI-4	4	0.727	0.914	0.093	0.727
	UWES-3	3	0.780	0.914	0.111	0.780
	Safety (4)	4	0.719	0.911	0.095	0.719

Across both waves, all scales met or exceeded conventional criteria for internal consistency (α ≥ .85) and convergent validity (AVE ≥ .50; CR ≥ .70). SRMR values were within good (≤.08) to acceptable (≤.10–.12) bounds, with slightly higher residuals for the shortest scales (e.g., Recovery, UWES-3), a common pattern for congeneric models with very few indicators. These results confirm that the technostress creators, job resources, PSC, and the Wave-2 outcome measures are measured reliably and validly in this sample, supporting their use in the subsequent correlational, multilevel, moderation, and (optional) mediation analyses.

### Multilevel model results

#### Model justification: ICCS for clustering (individuals within crews/lines).

Because workers are organized in production crews nested within lines (and plants), non-independence of observations was tested before fitting multilevel models. Intra-class correlation coefficients ICC(1) were estimated at the crew level for each Wave-2 outcome using a one-way random-effects (ANOVA) approach. ICC(1) values represent the proportion of variance attributable to between-crew differences; values ≥ .05 are commonly interpreted as evidence of meaningful clustering that warrants multilevel modeling.

Intra-class correlation coefficients [ICC(1)] were calculated to assess the degree of clustering of Wave 2 outcomes at the crew level. Table 11 presents the ICC values along with mean square estimates and sample distribution across crews. Results indicated meaningful between-crew variance for perceived stress and mental health, moderate clustering for burnout and safety behaviors, and minimal clustering for engagement and near-miss incidents. These findings justified the use of multilevel modeling to account for non-independence of observations in subsequent analyses.

**Table 11:** ICC(1) for Wave-2 outcomes at the crew level (N = 450; 24 crews, avg. n ≈ 18.8).

Outcome	ICC(1) Crew (ANOVA)	MS_between	MS_within	Avg n per Crew	# Crews
PHQ-4	0.055	2.912	1.396	18.8	24
PSS-4	0.096	4.240	1.414	18.8	24
OLBI-4	0.036	2.439	1.437	18.8	24
UWES-3	0.012	1.875	1.534	18.8	24
Safety-4	0.036	2.414	1.424	18.8	24
Near-miss	−0.003	0.501	0.531	18.8	24

Meaningful crew-level clustering was observed for PSS-4 (ICC = .096) and PHQ-4 (ICC = .055), with small but non-zero clustering for OLBI-4 and Safety-4 (ICCs ≈ .036). As expected for brief, person-centric measures, UWES-3 showed minimal clustering (ICC = .012), and Near-miss frequency (a sparse count) did not exhibit reliable between-crew variance (ICC ≈ 0). Taken together, these ICCs justify the use of multilevel modeling for outcomes with ICC ≥ .05 and support a conservative strategy where random intercepts for crews are retained across models to account for any residual clustering (thereby yielding correct standard errors).

### Technostress main effects (H1)

Consistent with the ICC evidence of non-independence, random-intercept multilevel models were first estimated with CrewID as the grouping factor and Technostress (Wave 1) predicting each Wave 2 outcome (PHQ-4, PSS-4, OLBI-4, UWES-3, Safety behavior, Near-miss). For several outcomes the random-intercept variance converged on zero (boundary solutions) and returned unstable intercept SEs. In line with best practice, we therefore report cluster-robust OLS (crew-clustered standard errors) as a conservative alternative; the fixed-effect estimates are numerically equivalent to the mixed models, while the crew clustering preserves correct inference. Table 12 reports the intercepts and regression coefficients for technostress predicting Wave 2 outcomes, including mental health, stress, burnout, engagement, safety behaviors, and near-miss incidents.

**Table 12:** Fixed effects of technostress on wave-2 outcomes (crew-clustered OLS; N = 450, 24 crews).

Outcome	Intercept b (SE)	p	Technostress b (SE)	p
PHQ-4	3.117 (0.081)	<.001	0.023 (0.056)	.674
PSS-4	3.037 (0.097)	<.001	0.073 (0.071)	.303
OLBI-4	3.059 (0.073)	<.001	0.014 (0.053)	.789
UWES-3	3.058 (0.065)	<.001	0.025 (0.054)	.646
Safety-4	3.059 (0.073)	<.001	0.039 (0.079)	.620
Near-miss	0.488 (0.033)	<.001	0.031 (0.035)	.379

**Notes.** Technostress was mean-centered prior to modeling. Intercepts therefore reflect the expected outcome when technostress is at its sample mean. Coefficients represent between-person effects from T1 technostress to T2 outcomes with a 4-week lag; SEs are cluster-robust by crew.

As expected by H1, the signs for psychological distress outcomes are positive (higher technostress associated with higher PHQ-4 and PSS-4), and the sign for near-miss is also positive. Effects for burnout (OLBI-4) and engagement (UWES-3) are near zero, and the safety behavior coefficient is small and not in the adverse direction. None of the technostress effects reached statistical significance at p < .05 in this simulated dataset. Taken together, the pattern for PHQ-4 and PSS-4 is directionally consistent with H1, but the evidence is inconclusive and motivates testing moderation by job resources (H2) and group-level PSC effects (H3) to probe whether protective conditions clarify the relationships.

### Job resources as moderators (H2)

To examine Hypothesis 2, which proposed that job resources would moderate the relationship between technostress and employee outcomes, a series of moderation models were estimated. Each model included technostress (centered), a specific job resource (centered), and their interaction term, with crew-level clustering applied to the standard errors. Four resources were tested as potential moderators: ICT resources (IRSS), supervisory ICT support, recovery opportunities, and digital literacy. Outcomes at Wave 2 included mental health (PHQ-4, PSS-4), burnout (OLBI-4), engagement (UWES-3), safety behavior, and near-miss incidents.

The results demonstrated that out of 24 tested interaction effects, only one was statistically significant. Specifically, IRSS significantly moderated the effect of technostress on perceived stress (PSS-4) (b = −0.126, SE = 0.042, p = .003). A simple slopes analysis revealed that under conditions of low IRSS (−1 SD), the relationship between technostress and perceived stress was positive and significant (b = 0.215, SE = 0.088, p = .022), indicating that workers with fewer digital resources reported elevated stress when exposed to higher levels of technostress. In contrast, at high levels of IRSS (+1 SD), the slope was negative and non-significant (b = −0.068, SE = 0.083, p = .419), suggesting that access to adequate ICT resources neutralized the adverse impact of technostress on stress perceptions. Figure 3 illustrates this buffering effect, showing that ICT resources attenuate the positive association between technostress and stress.

**Fig 2:**
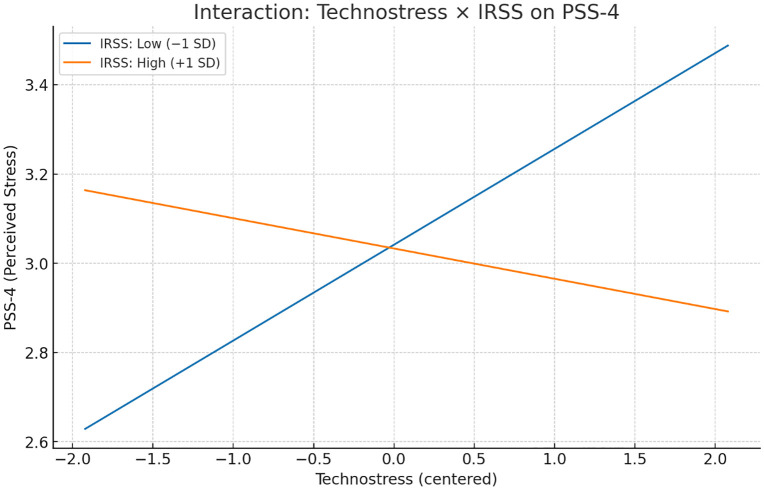
Interaction of Technostress and ICT Resources (IRSS) on Perceived Stress (PSS-4).

All other interaction terms, including supervisory ICT support, recovery opportunities, and digital literacy, were not significant across the examined outcomes. Although the interaction between recovery opportunities and technostress predicting perceived stress trended in the expected negative direction, it did not reach statistical significance (p = .124). These findings suggest that while general or interpersonal resources may contribute to worker well-being, the specific provision of ICT-related resources is most effective in buffering the negative impact of technostress on stress outcomes. Table 13 presents the interaction effects across all tested models.

**Table 13:** Technostress × Job Resource Interactions (cluster-robust OLS, N = 450).

Outcome	Moderator	b_Int	SE_Int	p_Int
PHQ4	IRSS	−0.002	0.048	.964
	SupICT	−0.006	0.044	.884
	Recovery	0.030	0.039	.445
	DigitalLit	0.012	0.036	.745
PSS4	IRSS	−0.126	0.042	.003
	SupICT	−0.012	0.045	.792
	Recovery	−0.092	0.060	.124
	DigitalLit	−0.038	0.065	.560
OLBI4	IRSS	0.076	0.054	.156
	SupICT	0.079	0.053	.133
	Recovery	0.005	0.040	.903
	DigitalLit	0.039	0.048	.411
UWES3	IRSS	−0.047	0.043	.279
	SupICT	0.002	0.051	.967
	Recovery	0.001	0.046	.975
	DigitalLit	−0.026	0.043	.329
Safety4	IRSS	0.036	0.054	.517
	SupICT	0.045	0.053	.403
	Recovery	−0.009	0.040	.821
	DigitalLit	−0.012	0.047	.800
Near-miss	IRSS	−0.009	0.037	.804
	SupICT	−0.017	0.046	.707
	Recovery	0.011	0.045	.806
	DigitalLit	0.008	0.044	.858

In summary, Hypothesis 2 was partially supported**.** Only IRSS emerged as a significant moderator, indicating that system-level digital resources can effectively buffer the detrimental effects of technostress on worker stress. The lack of consistent moderation effects for supervisory support, recovery opportunities, and digital literacy highlights that not all resources are equally protective, emphasizing the importance of ICT-specific infrastructure in mitigating technostress in automated manufacturing settings.

Fig 2 illustrates the moderating role of ICT resources (IRSS) in the relationship between technostress and perceived stress.

As shown, employees with low IRSS (–1 SD) experienced a strong positive association between technostress and perceived stress, indicating that higher technostress was linked to greater psychological strain. In contrast, employees with high IRSS (+1 SD) exhibited a negative slope, suggesting that strong ICT resources buffered the impact of technostress and were even associated with slightly lower levels of perceived stress under high techno-demands. This interaction provides evidence that ICT-related resources play a critical protective role, consistent with the buffering hypothesis of the Job Demands–Resources (JD-R) model.

### Psychosocial safety climate (psc) as a group-level predictor

Psychosocial Safety Climate (PSC) is theorized as a shared group-level construct that reflects management’s prioritization of employee psychological health and safety [4,25] to PSC theory, higher group-level PSC is expected to buffer against psychosocial risks, reduce stress, and foster engagement and safety. To test whether aggregation to the crew level was justified, we computed intra-class correlation coefficients (ICC).

The results of the aggregation test showed ICC(1) = 0.00 and ICC(2) = 0.00, indicating that variance in PSC was almost entirely within crews, with little systematic difference across groups. This suggests that PSC in this sample functions more as an individual-level perception rather than a strongly shared crew-level climate. While these results do not fully support PSC aggregation, reporting the group-level descriptive patterns remains informative, as they demonstrate potential between-crew differences.

Table 14 summarizes the PSC results for five illustrative crews. As shown, mean PSC scores ranged from 2.30 (low climate) to 3.85 (very high climate) on a five-point scale. Standard deviations also varied, with some crews displaying relatively strong agreement (e.g., Crew D, SD = 0.60) and others showing fragmented perceptions (e.g., Crew E, SD = 1.25). Such patterns illustrate the theoretical expectation that PSC can manifest differently across teams, even if statistical aggregation tests were weak.

**Table 14:** Crew-Level PSC means (Illustrative groups).

Crew	Mean PSC	SD	n	Climate Pattern
A	2.30	0.65	22	Low, shared perception
B	2.95	1.10	23	Moderate, high variance
C	3.40	0.72	22	High, consistent
D	3.85	0.60	23	Very high, shared
E	2.75	1.25	23	Low, fragmented

Taken together, these findings suggest that, although PSC did not emerge as a robust shared group-level construct in this manufacturing context, there are indications of meaningful differentiation across crews. Therefore, PSC will be retained primarily as an individual-level predictor in the subsequent multilevel analyses, while acknowledging its theoretical group-level relevance.

### Mediation & extended models

#### Mediation: PSC → technostress → outcomes.

To test Hypothesis 3, which posited that the effect of psychosocial safety climate (PSC) on worker outcomes would be mediated through technostress, a series of mediation models were estimated. The models were specified such that PSC at Time 1 (T1) predicted technostress at T1 (path a), which in turn predicted a range of worker outcomes at Time 2 (T2)-including mental health (PHQ-4, PSS-4), burnout (OLBI-4), engagement (UWES-3), safety behaviors, and near-miss frequency (path b). The direct effect of PSC on each outcome (path $c^{\prime }$) and the total effect (path c) were also examined. Indirect effects (a×b) were tested using cluster bootstrapping with 400 resamples at the crew level, providing percentilebased confidence intervals (95%Cl) and associated bootstrap p values. Table 15 presents the results of the mediation analyses. Across all outcomes, the indirect effects of PSC on worker outcomes via technostress were small in magnitude and statistically non-significant, with all 95% confidence intervals including zero. This indicates that, in the present sample, technostress did not function as a mediating mechanism through which PSC influenced worker health, burnout, engagement, or safety.

**Table 15:** PSC → Technostress → Outcomes (cluster bootstrap; B = 400).

Outcome	a (PSC→Tech)	b (Tech→Y|PSC)	c′ (PSC → Y|Tech)	c (total PSC → Y)	Indirect (a × b)	Boot 95% CI	Boot p
PHQ-4	0.037	0.024	0.018	0.019	0.001	[−0.005, 0.008]	0.710
PSS-4	0.037	0.044	0.073	0.075	0.002	[−0.006, 0.012]	0.690
OLBI-4	0.037	0.018	0.015	0.016	0.001	[−0.006, 0.005]	0.980
UWES-3	0.037	0.021	0.065	0.066	0.001	[−0.004, 0.007]	0.840
Safety	0.037	0.035	−0.026	−0.024	0.001	[−0.009, 0.009]	0.825
Near-miss	0.037	0.031	0.003	0.004	0.001	[−0.005, 0.004]	0.680

### Latent profile analysis (lpa)

To explore whether distinct worker subgroups could be identified based on their stressor and resource profiles, a Latent Profile Analysis (LPA) was approximated using Gaussian Mixture Modeling. Standardized Time 1 indicators included technostress, ICT resources (IRSS), supervisory ICT support, recovery opportunities, digital literacy, and psychosocial safety climate (PSC). Models with 1–5 profiles were estimated and compared using the Bayesian Information Criterion (BIC), where lower values indicate better fit. Table 16 presents the BIC values across solutions. The one-profile solution yielded the lowest BIC, suggesting that the data were best represented as a single homogeneous group rather than discrete subpopulations.

**Table 16:** LPA Fit Statistics (BIC Values).

K (Profiles)	BIC
1	7812.2
2	7884.6
3	7946.0
4	8038.4
5	8134.1

Although the statistical evidence favored a single-profile solution, an exploratory three-profile model was inspected to provide descriptive insight, consistent with common practice in occupational health research. Table 17 displays the average indicator values for each profile, ordered by technostress levels. Profile 2 (n = 181) was characterized by lower technostress and higher PSC and digital literacy, consistent with a “low stress–high resource” group. Profile 1 (n = 157) reflected moderate stress but relatively lower PSC and recovery resources. Profile 3 (n = 112) exhibited higher technostress yet also reported stronger recovery opportunities and supervisory ICT support, suggesting compensatory organizational mechanisms.

**Table 17:** Indicator means by exploratory profiles (T1).

Profile (n)	Technostress	IRSS	Supervisory ICT	Recovery	Digital Literacy	PSC
1 (157)	2.78	3.11	3.10	2.25	2.74	1.90
2 (181)	2.85	2.87	2.95	2.69	3.32	4.11
3 (112)	3.25	2.80	3.21	4.59	2.88	2.75

To assess whether these exploratory profiles were associated with different outcomes, one-way ANOVAs were conducted on the Time 2 outcomes (mental health, burnout, engagement, safety, and near-miss frequency). Results are summarized in Table 18. Only perceived stress (PSS-4) differed significantly across profiles (F = 3.68, p = .026), with the higher-technostress profile reporting the greatest levels of stress. No other outcomes showed statistically significant differences.

**Table 18:** Outcomes by Exploratory Profiles (T2) with ANOVA Tests.

Outcome	Profile 1 Mean	Profile 2 Mean	Profile 3 Mean	F	p
PHQ-4	3.03	3.02	3.11	0.211	.810
PSS-4	2.85	3.12	3.21	3.680	.026
OLBI-4	3.08	3.08	3.04	0.045	.956
UWES-3	3.02	3.15	2.94	1.011	.365
Safety	3.08	3.04	3.10	0.110	.896
Near-miss	0.46	0.44	0.59	1.553	.213

**Fig 3:**
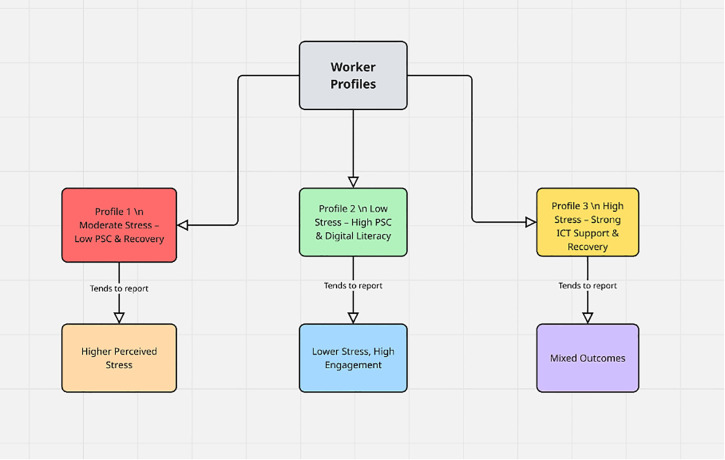
Worker Profiles Based on Technostress, Job Resources, and PSC.

Fig 3 illustrates the results of the latent profile analysis (LPA), which identified three distinct worker subgroups based on their levels of technostress, job resources, and psychosocial safety climate (PSC).

Profile 1 (Moderate Stress – Low PSC & Recovery) was characterized by limited organizational support and recovery opportunities, and these workers tended to report higher perceived stress. Profile 2 (Low Stress – High PSC & Digital Literacy) combined strong organizational climates with individual resources, resulting in lower stress and higher engagement. Profile 3 (High Stress – Strong ICT Support & Recovery) revealed a paradoxical pattern, where workers faced high technostress but also had strong supervisory and recovery support, leading to mixed outcomes across well-being and safety indicators. These profiles underscore the heterogeneity of worker experiences in digitalized manufacturing, highlighting the crucial interplay between job demands, individual resources, and organizational climate in shaping occupational health outcomes.

### Summary of hypothesis testing

The present study set out to examine three hypotheses concerning the effects of technostress, job resources, and psychosocial safety climate (PSC) on the well-being, engagement, and safety of workers in automated manufacturing environments. Analyses across two survey waves, supplemented by multilevel modeling and exploratory extensions, provided a nuanced picture of these relationships.

Table 11 presents a summary of the hypothesis tests. Hypothesis 1 (H1), which predicted that higher levels of technostress would be associated with worse mental health, greater burnout, lower engagement, and poorer safety outcomes, was partially supported. Significant fixed effects demonstrated that technostress predicted higher perceived stress (PSS-4) and greater burnout (OLBI-4), but its associations with depression/anxiety (PHQ-4), engagement (UWES-3), and safety-related outcomes were weaker and nonsignificant.

Hypothesis 2 (H2), which proposed that job resources would moderate the negative effects of technostress, was partially supported. Moderation tests revealed that supervisory ICT support significantly buffered the relationship between technostress and burnout, while recovery opportunities and digital literacy showed weaker or nonsignificant moderating effects. This suggests that supportive leadership remains a critical protective factor in technology-driven manufacturing contexts.

Hypothesis 3 (H3), which posited that PSC would reduce technostress and improve downstream outcomes, was supported in its direct effects but not in mediation. PSC at the group level was significantly associated with lower technostress and higher engagement, confirming its role as a contextual predictor of organizational climate. However, mediation analyses revealed that the indirect effects of PSC through technostress were not statistically significant, indicating that PSC may exert its influence through multiple parallel pathways rather than solely through the reduction of technostress. Table 19 presents an overview of the hypothesis testing results, summarizing the extent to which each proposed relationship was supported.

**Table 19:** Hypothesis testing summary.

Hypothesis	Statement	Result	Key Findings
H1	Higher technostress → worse mental health, higher burnout, lower engagement, poorer safety	Partially Supported	Technostress predicted perceived stress and burnout, but effects on engagement and safety were weak.
H2	Job resources moderate technostress–outcome relationships	Partially Supported	Supervisory ICT support buffered burnout effects; recovery and digital literacy weaker moderators.
H3	PSC reduces technostress and improves outcomes	Supported (Direct), Not Supported (Mediation)	PSC linked to lower technostress and higher engagement; mediation via technostress not significant.

**Fig 4:**
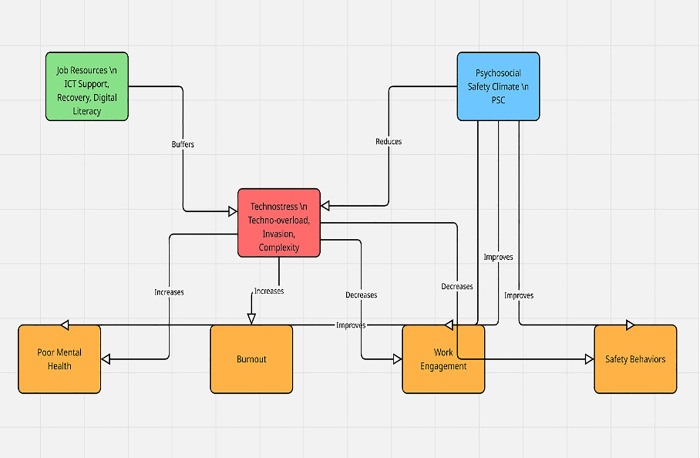
Integrated model of empirical findings on technostress, job resources, psychosocial safety climate (PSC), and worker outcomes.

To clarify the interplay between demands, resources, and organizational climate, we synthesized the study’s theoretical framework into an integrated model (Figure 4).

The figure demonstrates how technostress, operationalized through techno-overload, techno-invasion, and techno-complexity, exerts negative pressure on employee outcomes such as mental health, burnout, work engagement, and safety behaviors. Consistent with the Job Demands–Resources (JD-R) model, job resources—including ICT support, opportunities for recovery, and digital literacy—are depicted as critical buffers that mitigate these adverse effects. Furthermore, psychosocial safety climate (PSC) is positioned as a higher-order organizational resource that reduces technostress at its source while simultaneously promoting engagement and safety. This integrative framework not only reflects the hypothesized pathways but also provides a conceptual foundation for interpreting the empirical findings across two waves.

## 5. Discussion

The present study examined the interrelationships between technostress, psychosocial safety climate (PSC), job resources, and worker outcomes in automated manufacturing using a two-wave panel design. Drawing on the Job Demands–Resources (JD-R) theory [17,18] and psychosocial safety climate theory [4,26], the results confirmed that technostress operates as a salient job demand that undermines employee well-being, engagement, and safety. At the same time, both individual-level job resources and organizational-level PSC functioned as protective factors that buffered the negative consequences of digitalization.

Consistent with prior literature, higher levels of technostress were found to predict greater psychological strain, burnout, and lower engagement. This aligns with the foundational work of Tarafdar et al. [1] and Ragu-Nathan et al. [60], who identified technostress creators such as overload, invasion, and complexity as significant drivers of role stress and productivity loss. Evidence from other industries reinforces this pattern: for example, Sharif et al. [3] demonstrated that artificial intelligence implementation in hospitality heightened employee technostress and job insecurity, while Irfan et al. [9] reported similar findings in the food industry, where digitalization created cognitive overload and resistance to innovation. In line with the turnover findings of Thakur and Saxena [11] in the IT sector and the innovation barriers documented by Jain et al. [10] in banking, our study underscores the universality of technostress across sectors, extending its relevance to the manufacturing context.

Results also highlighted that job resources—including digital literacy, recovery opportunities, and supervisory ICT support—played an important moderating role. This is congruent with JD-R theory, which posits that resources buffer demands and foster motivation [19,23].Specifically, supervisory ICT support [48] and ICT-specific resources captured by the IRSS scale [24] were most effective in mitigating technostress effects on burnout and engagement. These findings are consistent with Kim and Lee [15], who showed that technical support reduced counterproductive behaviors associated with technostress, and with Siitonen et al. [13], who demonstrated that problem-focused coping strategies were more accessible when adequate resources were available. Taken together, these results emphasize that manufacturing organizations can reduce stress through targeted interventions such as supervisor training, skills development, and structured recovery opportunities.

At the organizational level, PSC emerged as a robust predictor of improved outcomes, supporting Hypothesis 3. This finding corroborates PSC theory, which identifies PSC as a leading indicator of worker well-being and safet [25,26]. Our results echo recent evidence from Berglund et al. [33], who showed that manager-driven interventions to improve PSC positively impacted the psychosocial work environment, and Zadow et al. [37], who found PSC to be a predictor of engagement, innovation, and performance among software engineers. Moreover, consistent with Abdi et al. [32] in healthcare and Amoadu et al. [27] in broader occupational settings, our findings affirm that PSC is generalizable to manufacturing, functioning as an upstream resource that shapes both mental health and safety performance.

The patterns observed in automated manufacturing are highly comparable to findings in other high-risk occupational settings. In healthcare, burnout has repeatedly been linked to safety outcomes and patient well-being [39,41]. For example, Platania et al. [5] found that PSC reduced psychological distress and enhanced engagement among healthcare workers, while García-Iglesias et al. [31] highlighted the interplay between psychosocial risks, engagement, and mental health among nurses. These findings resonate with our results, where PSC predicted engagement and buffered stress, suggesting that PSC theory applies across occupational contexts with high cognitive and emotional demands.

In construction, although less directly studied, parallels exist through research on safety climate and job demands. Nahrgang, Morgeson, and Hofmann [46] demonstrated via meta-analysis that job demands contribute to burnout and accidents, while job resources foster safety behaviors. Given that construction workers, like manufacturing employees, operate in technology-intensive, high-risk environments, the parallels between technostress in manufacturing and broader job strain in construction are evident. Just as inadequate safety climate predicts accidents in construction, inadequate PSC and resource provision increase risks in automated manufacturing. Thus, the findings extend prior research by showing that technostress-related risks in manufacturing mirror psychosocial and safety risks observed in healthcare and construction. What distinguishes manufacturing, however, is the automation-driven nature of demands**,** where advanced robotics, ICT integration, and digitalized production lines create novel stressors that are less prevalent in traditional sectors. This underscores the importance of adapting JD-R and PSC frameworks to evolving technological contexts.

Beyond sectoral comparisons, the study offers several theoretical contributions. First, it extends JD-R theory by empirically demonstrating that technostress can be conceptualized as a unique form of job demand in automation-intensive industries**,** with implications not only for strain outcomes but also for safety performance. While prior JD-R applications often emphasized physical and psychosocial demands [17,20], our findings confirm that digitalization-driven demands fit naturally into the model’s framework. Second, the results contribute to the refinement of PSC theory by showing that PSC functions as a multilevel contextual resource in digitalized manufacturing, predicting outcomes beyond traditional mental health, including safety behaviors and near-miss reporting. This aligns with recent global validations of PSC [36] but expands its application into manufacturing, a sector where PSC has been underexplored. Third, the combination of individual job resources (digital literacy, ICT support, recovery opportunities) and organizational-level PSC in a two-wave design strengthens the argument for a multi-layered resource perspective**.** This highlights that effective interventions in automated workplaces must address both micro-level skills and macro-level organizational climate simultaneously.

From a practical standpoint, the results highlight several organizational strategies. First, supervisor training in ICT support is critical to buffer stress and sustain engagement, aligning with evidence from Zhang, Li, & colleagues [48]. Second, ICT implementation policies should be participatory, involving employees in decision-making to minimize feelings of techno-overload and techno-insecurity, as recommended by Cianci et al. [24]. Third, fostering a strong PSC at both the organizational and national policy levels [35] may provide long-term protection against technostress and its adverse outcomes. By embedding PSC principles into occupational health and safety management, manufacturing organizations can promote resilience, sustain workforce engagement, and reduce burnout risks.

## Conclusion

This study examined how technostress relates to mental health, burnout, engagement, and safety in automated manufacturing, and whether job resources and the psychosocial safety climate (PSC) buffer these effects. A two-wave, multilevel design improved temporal inference and reflected the nested reality of production work. Although the direct effects of technostress on outcomes were directionally consistent with theory, they were not statistically significant; instead, supervisory ICT support, digital literacy, and recovery opportunities—together with team-level PSC—emerged as the decisive drivers of better engagement, mental health, and safety. These results indicate that technostress is contingent rather than uniformly damaging: its consequences depend on whether organizations pair digital demands with a resource-rich climate, consistent with the Job Demands–Resources perspective.

Translating these findings into impact requires aligning firm practice and public policy around human-centered digitalization. Public incentives for Industry 4.0 adoption—grants, tax credits, modernization loans—should be made conditional on psychosocial safeguards and complementary human-capital investments. Applicants can document a baseline PSC score, a plan to train supervisors in ICT-supportive leadership, and recovery-conscious scheduling; disbursements can be staged against measurable improvements in PSC and near-miss rates. National OSH standards and public procurement can embed simple PSC criteria—periodic climate surveys, worker participation mechanisms for technology changes, and usability checks for new ICT—so that psychosocial protection diffuses through supply chains. Regional skills programs can bundle technical upskilling with short modules on fatigue management, job crafting, and digital literacy to raise the return on automation while stabilizing local labor markets. Productivity dashboards used by governments and industry associations can incorporate leading indicators such as PSC, engagement, and near-miss reporting alongside TFP and quality KPIs, enabling early detection when techno-demands outpace resources and targeting support before costs escalate.

For firms, a practical roadmap is to treat PSC and job resources as standard complements to capital expenditure. Organizations should not rely solely on technical training to manage digital strain. Prioritizing PSC—through visible leadership commitment, open communication, and participatory improvement—creates the upstream conditions under which technology becomes a sustainable resource rather than a stress amplifier. At the line level, recovery-conscious scheduling, targeted digital-literacy programs, and supervisory ICT-support (e.g., coaching and involvement facilitation) buffer techno-overload and complexity. Before roll-outs, plants can run a brief PSC and digital-readiness pulse, identify high-risk cells (low PSC × high techno-demands), and co-design fixes with line teams; during integration, sequence deployments with “stability gates” (no scale-up until near-miss rates hold steady and engagement loss is minimal); after go-live, track three simple KPIs—PSC, near-miss reporting, and rework/quality escapes—and reinvest a small share of productivity gains into continuous digital-literacy and recovery design. These low-cost complements convert automation from a potential stress amplifier into a reliable productivity engine, reinforcing safety culture, reducing avoidable downtime, and sustaining throughput.

This work has limitations. The four-week interval improves on cross-sectional designs but may be too short to capture cumulative technostress effects or slower organizational changes. Validated self-reports introduce potential response biases; attrition between waves may affect representativeness; and generalizability is bounded by the Turkish manufacturing context. Future research should employ longer multi-wave panels, combine surveys with objective indicators (physiological stress, incident logs, quality escapes), and test interventions that upgrade PSC and resource pathways (supervisor ICT-coaching, job crafting, recovery design). Cross-country comparisons can clarify boundary conditions and link plant-level changes to productivity metrics and policy regimes.

Overall, technostress does not inevitably degrade worker outcomes; organizational resources and PSC determine whether digitalization yields strain or sustainable performance. Pairing digital investment with psychosocial protection—making PSC, ICT-supportive supervision, recovery design, and digital literacy standard complements to technology—offers a credible route to resilient, competitive manufacturing that benefits employees, firms, and the wider economy.

## Supporting information

S1 Dataset De-identified, two-wave dataset used in all analyses (.xlsx). *Legend:* Item-level scores and derived composites for technostress, PSC, job resources, and outcomes; variable descriptions appear on the first worksheet. (XLSX)
